# Comparisons between artificial intelligence computer-aided detection synthesized mammograms and digital mammograms when used alone and in combination with tomosynthesis images in a virtual screening setting

**DOI:** 10.1007/s11604-022-01327-5

**Published:** 2022-09-07

**Authors:** Takayoshi Uematsu, Kazuaki Nakashima, Taiyo Leopoldo Harada, Hatsuko Nasu, Tatsuya Igarashi

**Affiliations:** 1 Department of Breast Imaging and Breast Intervention Radiology, Shizuoka Cancer Center Hospital, Shizuoka, Japan; 2 Department of Radiology, Hamamatsu University School of Medicine, Shizuoka, Japan; 3 Department of Radiology, Fujieda Municipal General Hospital, Shizuoka, Japan

**Keywords:** Synthetic mammography, Digital breast tomosynthesis, Digital mammography, Artificial intelligence, Breast cancer

## Abstract

**Purpose:**

To compare the reader performance of artificial intelligence computer-aided detection synthesized mammograms (AI CAD SM) with that of digital mammograms (DM) when used alone or in combination with digital breast tomosynthesis (DBT) images.

**Materials and methods:**

This retrospective multireader (*n* = 4) study compared the reader performances in 388 cases (84 cancer, 83 benign, and 221 normal or benign cases). The overall accuracy of the breast-based assessment was determined by four radiologists using two sequential reading modes: DM followed by DM + DBT; and AI CAD SM followed by AI CAD SM + DBT. Each breast was rated by each reader using five-category ratings, where 3 or higher was considered positive. The area under the receiver-operating characteristic curve (AUC) and reading time were evaluated.

**Results:**

The mean AUC values for DM, AI CAD SM, DM + DBT, and AI CAD SM + DBT were 0.863, 0.895, 0.886, and 0.902, respectively. The mean AUC of AI CAD SM was significantly higher (*P* < 0.0001) than that of DM. The mean AUC of AI CAD SM + DBT was higher than that of DM + DBT (*P* = 0.094). A significant reduction in the reading time was observed after using AI CAD SM + DBT when compared with that after using DM + DBT (*P* < 0.001).

**Conclusion:**

AI CAD SM + DBT might prove more effective than DM + DBT in a screening setting because of its lower radiation dose, noninferiority, and shorter reading time compared to DM + DBT.

## Introduction

Digital breast tomosynthesis (DBT) images have been successfully used for population-based breast cancer screening. There is evidence of improved overall performance with digital mammograms (DM) when used along with DBT compared to that when used alone [1]. However, the use of DM + DBT implies a twofold increase in the radiation dose, which is not acceptable for the screening of asymptomatic women. Synthesized mammograms (SM) consist of two-dimensional images that are generated from the DBT data to prevent exposure to additional radiation doses; SM + DBT have been used to replace DM + DBT for the screening process [1–3]. However, DBT is commonly used in combination with DM or SM. This increases in the reading time because the radiologist has to read many slice images generated by the DBT data [4], which could act as a barrier for the implementation of DBT in screening.

In the recent years, deep learning-based artificial intelligence (AI) systems have been quickly evolving in the field of breast imaging and surpassing the performance and clinical value of traditional computer-aided detection (CAD) for mammography [5]. In addition, the use of an AI CAD-enhanced SM enabled radiologists to review the DBT 23.5% at a faster rate, without altering the interpretation performance, when compared to that without CAD [6]. Previous studies have reported that the use of AI CAD SM can automatically enhance suspicious findings in the DBT data, thereby improving the quality of the SM [7–9]. We hypothesized that newer versions of SM using AI CAD-like technology could improve the quality of images and enhance the performance of the radiologists along with a reduction in the reading time. The aim of this study was to assess the reader performance of new SM and compare it with that of the original full-field DM; the comparisons were made using the mammograms alone or in combination with DBT in a virtual screening setting.

## Materials and methods

### Study design

This retrospective, fully crossed, completely randomized, multireader, multicase study was approved by the Institutional Review Board of our hospital. Written informed consent to was obtained from all the recruited patients enrolled in this study. FUJIFILM (Tokyo, Japan), which invented the new SM for the reading, provided the equipment and support for this study. The data and materials submitted for publication were always under the control of the researchers who were not FUJIFILM personnel. According to the joint research and development agreement, 500 DM + DBT examinations with a bilateral two view (cranio-caudal/mediolateral oblique CC/MLO) image were performed at ? Hospital between January 2020 and August 2020. All DM + DBT examinations were performed with a narrow-angle DBT system with 15-degree tube motion from 15 projection images (AMULET Innovality; FUJIFILM)). DM and DBT were performed during a single breast compression per view. We decided that the comparison of the reader performance was performed at the per-breast level using only mediolateral oblique (MLO) view images taking the reading time and readers’ attention span during the reading study into consideration.

### Case selection

We excluded patients with past surgical history from a case set and developed the case set of 388 breast images from 198 DM/AI CAD SM + DBT examinations in sequence based on the diagnostic work-up patients, which comprises 84 breast cancers, 83 biopsy-proven benign lesions, and 221 normal or benign cases (BI-RADS score of 1 or 2) [10] with negative results after 1 year follow-up. We included dataset with anonymized images and patient’s data with pathology results if an image-guided biopsy and/or surgery was performed. The distribution and mammographic findings were 83 masses (49 malignant and 34 benign), 16 masses with associated calcifications (15 malignant and 1 benign), 5 focal asymmetry densities (2 malignant and 3 benign), 10 architectural distortions (4 malignant and 6 benign), and 53 calcifications (14 malignant and 39 benign). The lesion size of the malignant cases with mass type, ranged from 6 to 42 mm (median, 19 mm; mean, 20 mm). The verified cancer cases included 71 cases of invasive ductal carcinoma, 8 cases of DCIS, 3 cases of invasive lobular carcinoma, 1 case of tubular carcinoma, and 1 case of invasive micropapillary carcinoma. The breast density distribution ratings of the cases were 206 of 388 (53%) non-dense breasts (including almost entirely fatty or scattered fibroglandular densities) and 182 of 388 (47%) dense breasts (including heterogeneously dense or extremely densities), indicating the tissue density BI-RADS score [10]. The age of the study cohort ranged from 23 to 88 years (median, 52 years; mean, 54 years).

### Generation of new SM

We utilized the latest version (under development) of FUJIFILUM image processing approach to generate the AI CAD SM used in this study. The AI algorithm uses a fast and efficient convolutional neural network, ESPNet [11]. They have improved different finding patterns, such as soft tissue densities, linear structures, and calcifications, detected and extracted from DBT data using AI CAD-like technology. They are new pixel-based synthesis algorithm that merges the selected DBT plane based on the AI CAD detections from the DBT data. The lesions detected by AI CAD are not marked or outlined on AI CAD SM.

### Image readers

Four Japanese radiologists who were ranked as having an “A” certification from the Japan Central Organization on Quality Assurance of Breast Cancer Screening (i.e., achieved the scores required for a DM reading instructor in Japan) performed the image evaluations. The radiologists were blinded to the case selection. The readers had between 5 and 30 years of experience in mammography (median, 12 years) and their experience with DBT ranged from 2 to 8 years (median, 4 years). None of the radiologists had prior experience with the new SM reading.

### Evaluation methods

The evaluations of the images by the readers were conducted in two stages. The examinations were read twice as follows: (a) DM followed by DM + DBT, and (b) AI CAD SM followed by AI CAD SM + DBT. At least 4 week intervals were allowed as wash-out periods. In each reading mode, the radiologists were prompted to rate each breast using the two-dimensional (2D) image alone followed by the rating of the DBT using the five-point forced BI-RADS (1, 2, 3, 4, or 5) scores [10]. The most suspicious finding was marked and rated on the electronic reporting system.

The reading times were automatically recorded per examination using the workstation software from the moment the examination was displayed until the reader document the image findings including the locations and radiological characterizations of the cancers and benign lesions were documented. The readers did not use a timer and were blinded to the reading time.

To acquaint themselves with the workstation and interpretation procedure, all radiologists were trained to review sets of 100 DM + DBT and sets of 200 breast images from 100 DM/AI CAD SM + DBT examinations (not included in the study) before participating in this study.

The radiologists used a reading dual-monitor workstation with an electronic reporting system (Climb-Mammography WS; Climb Medical Systems) and a 5MP DB/DBT-certified diagnostic color display (CCL-S500; JVCKENWOOD) calibrated to the DICOM Grayscale Standard Display Function, for the image interpretation.

### Reference standard

An unblinded review of every case per breast was performed by one radiologist (?, a breast imaging radiologist with 30 years of experience in mammogram interpretation and 10 years of experience in tomosynthesis image interpretation), who was not involved in the observer study, to determine　the standard pathologic reference and reference mammographic findings. The radiologist also confirmed that there were no discrepancies between the pathologic diagnoses and mammographic findings of 84 breast cancers and 83 biopsy-proven benign lesions. The number of true and false interpretations by the readers was assessed for each image, including the location and radiological characterization of the cancers and benign lesions and the confirmed normal status. This study was modeled in a screening setting: hence, the final assessments of the images were categorized as follows: positive (BI-RADS score, 3–5) and negative (BI-RADS score, 1–2).

### Statistical analysis

The mean area under the receiver-operating characteristic (ROC) curve (AUC) was used to assess the accuracy of the reported forced BI-RADS ratings. The reader-averaged AUC was analyzed using the random-reader, random-case model to account for the possible correlations during the evaluation of the breasts in the same patient. Additionally, the correlations between repeated assessments of the same cases by different radiologists using different modalities, and between-reader variabilities were assessed. Bootstrap percentile confidence intervals (CI) and the corresponding estimated *P* values were computed based on the 100,000 bootstrap samples (1000 resamples of cases by 100 resamples of readers). A linear mixed model with the subject and reader as variable factors was used for inter-method comparisons of the reading time. Statistical inferences for all analyses were performed at a significance level of 0.05. The statistical analyses were performed using the R package for Windows (version 4.0.2, R Foundation for Statistical Computing, Vienna, Austria).

## Results

The mean (± standard deviation) compressed breast thickness during DM + DBT was 38.8 mm ± 14.5. The mean glandular dose for a single mammographic view was 1.12 mGy ± 0.32 (standard deviation) for DM and 1.62 mGy ± 0.49 for DBT. These dose levels constitute an average dose reduction of 41% (1.12 mGy/2.74 mGy) for AI CAD SM + DBT as compared with DM + DBT.

**Fig. 1:**
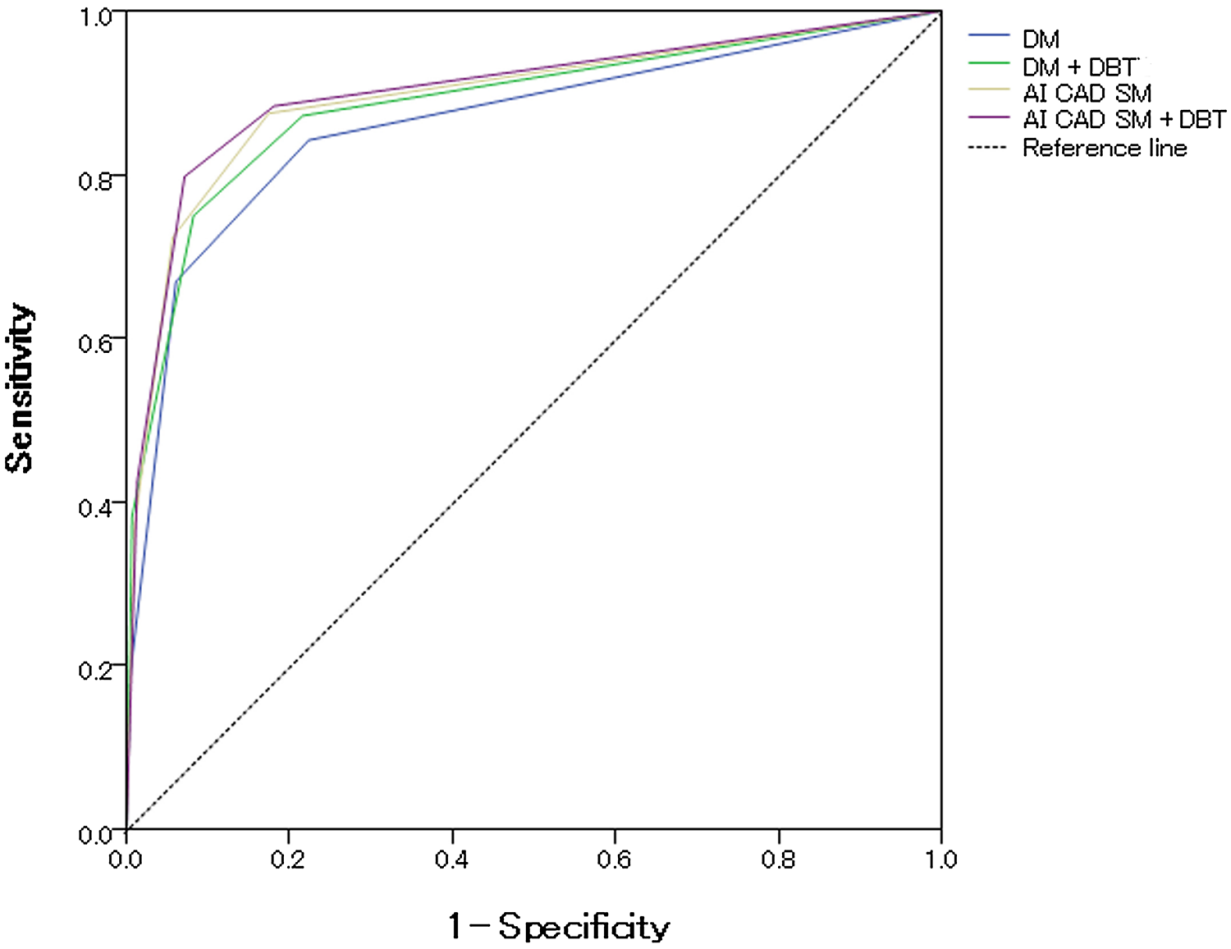
The overall ROC curve based on the five-point forced BI-RADS scores ratings for each case

The mean AUC values for DM, AI CAD SM, DM + DBT, and AI CAD SM + DBT were 0.863, 0.895, 0.886, and 0.902, respectively. The AUC of AI CAD SM alone was significantly higher than that of DM alone (difference, 0.033; 95% CI 0.013, 0.054; *P* < 0.0001; Fig. 1). All four readers performed somewhat better with AI CAD SM than with DM (Table 1). The AUC of AI CAD SM + DBT was higher than that of DM + DBT (difference, 0.016; 95% CI − 0.003, 0.035; *P* = 0.094), and three readers performed somewhat better with AI CAD SM + DBT than with DM + DBT. In addition, the mean AUC of AI CAD SM alone and DM + DBT did not differ significantly (*P* = 0.312); and three readers performed somewhat better with AI CAD SM alone than with DM + DBT. Except for a single reader, who performed slightly worse (AUC difference, 0.006) with DM + DBT when compared with DM alone, improvements in the performances of the remaining three readers were observed when DBT was made available to them after a review of either the DM or AI CAD SM alone.

**Table 1:** AUC based on the forced bi-rads ratings from each reader for the breast-level analysis

Reader	AUC-based forced BI-RADS ratings			
	DM	SM	DM + DBT	SM + DBT
1	0.858	0.906	0.889	0.913
2	0.885	0.893	0.914	0.898
3	0.838	0.908	0.886	0.920
4	0.869	0.878	0.863	0.885
Mean	0.863	0.895	0.886	0.902

*DM* digital mammograms, *SM* synthesized mammograms, *DBT* digital breast tomosynthesis images

**Fig. 2:**
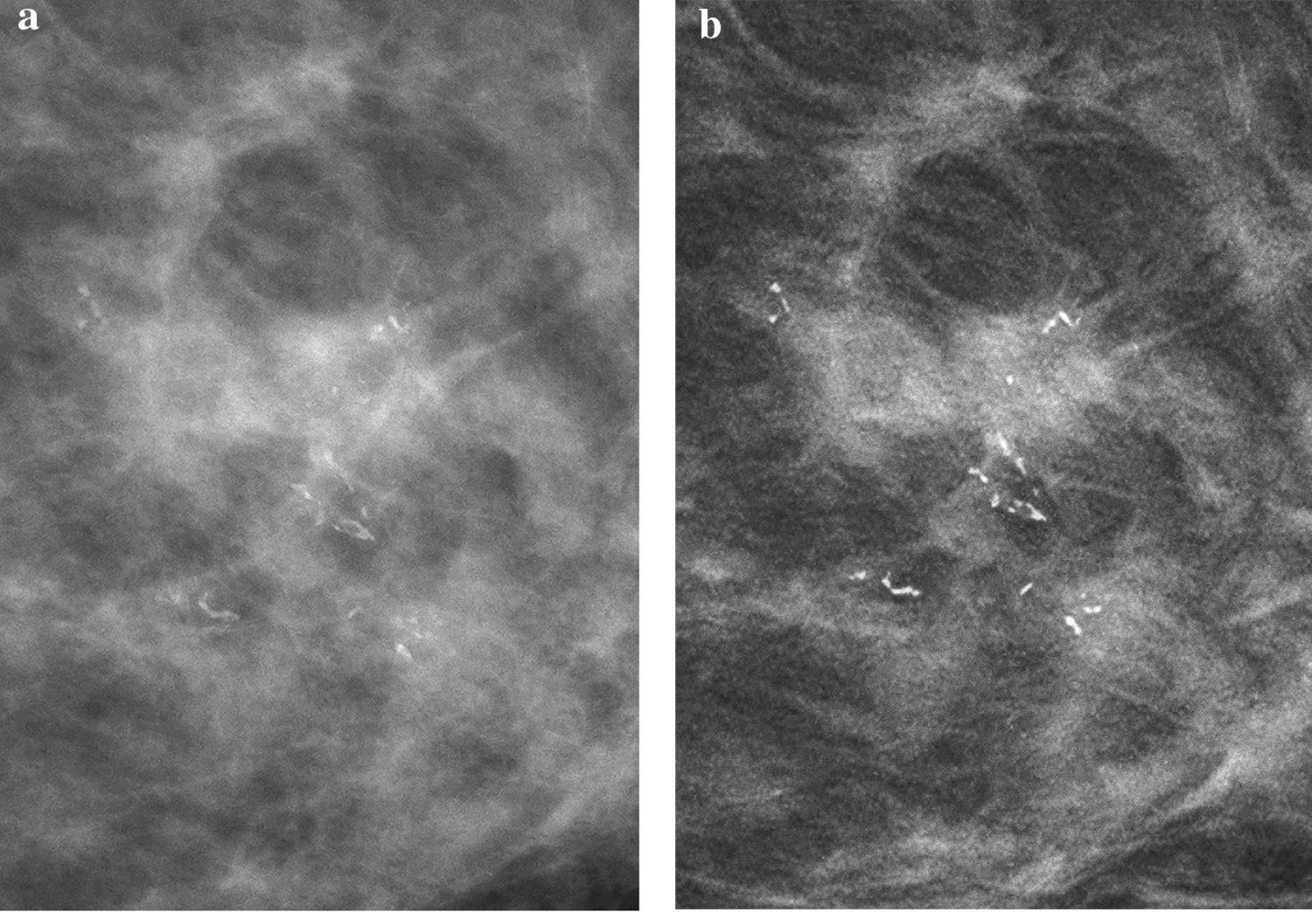
Image of a 46-year-old woman with scattered fibroglandular breasts, which was confirmed as an invasive ductal carcinoma during surgery. **a** DM shows the presence of grouped fine linear calcifications (24 mm); the associated mass (10 mm) is not clear. **b** SM clearly demonstrates the grouped fine linear calcifications with an associated speculated mass. The visualization is better than that with the DM

The cancer detection sensitivities for DM, AI CAD SM, DM + DBT, and AI CAD SM + DBT were 84.2, 87.5, 87.2, and 88.4%, respectively (Table 2). The detection sensitivity was improved after using AI CAD SM alone compared to DM alone (difference, 0.033; 95% CI − 0.003, 0.068; *P* = 0.056; Fig. 2). No significant difference in sensitivity was observed between AI CAD SM alone and DM + DBT *(P* = 0.173), whereas a significant difference was noted between DM alone and DM + DBT (*P* = 0.034). In the dense breast cases (*n* = 32), the sensitivities of the 2D images alone were improved in the corresponding sequential reading modes when DBT were made available to them (after DM: difference, 0.055; 95% CI 0.000, 0.109; *P* = 0.034; after AI CAD SM: difference, 0.031; 95% CI 0.008, 0.063; *P* < 0.0001).

**Table 2:** Sensitivities of each reading mode for the malignant lesions and each finding

	DM (%)	SM (%)	DM + DBT (%)	SM + DBT (%)
Cancer detection (*n* = 84)	84.2	87.5	87.2	88.4
Non-dense breast (*n* = 52)	88.0	90.9	89.4	90.4
Dense breast (*n* = 32)	78.1	82.0	83.6	85.2
Calcification lesion (*n* = 14)	96.4	92.9	96.4	92.9

*DM* digital mammograms, *SM* synthesized mammograms, *DBT* digital breast tomosynthesis

For the malignant calcification lesions (*n* = 14), the sensitivities for DM, AI CAD SM, DM + DBT, and AI CAD SM + DBT were 96.4, 92.9, 96.4, and 92.9%, respectively (Table 2). No significant differences were observed between the sensitivities of AI CAD SM and DM alone (*P* = 0.286) and DM + DBT and AI CAD SM + DBT (*P* = 0.286).

Similarly, for the verified benign cases (*n* = 83), the sensitivities for DM, AI CAD SM, DM + DBT, and AI CAD SM + DBT were 53.6, 47.3, 55.7, and 48.8%, respectively (Table 3). Significant differences were observed between DM and AI CAD SM alone (*P* = 0.012), DM + DBT and AI CAD SM + DBT (*P* = 0.002), and AI CAD SM alone and DM + DBT (*P* = 0.001). These findings indicated that the benign recall rates of AI CAD SM were lower than those of DM. The sensitivity of AI CAD SM alone was significantly lower than that of DM + DBT in the dense breast cases.

**Table 3:** Sensitivities of each reading mode for benign lesions and each finding

	DM (%)	SM (%)	DM + DBT (%)	SM + DBT (%)
Verified benign lesion detection (*n* = 83)	53.6	47.3	55.7	48.8
Non-dense breast (*n* = 42)	54.2	45.2	53.6	44.6
Dense breast (*n* = 41)	53.0	49.4	57.9	53.0
Calcification lesion (*n* = 39)	44.9	31.4	44.2	30.8

*DM* digital mammograms, *SM* synthesized mammograms, *DBT* digital breast tomosynthesis images

The sensitivities for DM, AI CAD SM, DM + DBT, and AI CAD SM + DBT in the benign calcification lesions (*n* = 39) were 44.9, 31.4, 44.2, and 30.8%, respectively (Table 3). The sensitivity of AI CAD SM alone was significantly lower than that of DM alone (*P* = 0.001). Similarly, the sensitivity of AI CAD SM + DBT was significantly lower than that of DM + DBT (*P* < 0.0001), and that of AI CAD SM alone was significantly lower than that of DM + DBT (*P* = 0.002).

The mean reading time per examination for AI CAD SM + DBT (91.87 s; 95% CI 70.46–113.27 s) was significantly shorter (*P* < 0.001) than that of DM + DBT (99.08 s, 95% CI 77.67–120.48 s; Table 4). Alternatively, the reading time was shorter using DM alone (19.95 s; 95% CI 19.95–62.77 s) when compared to that using AI CAD SM alone (42.37 s; 95% CI 20.96–63.78 s), statistical significance notwithstanding (*P* = 0.555).

**Table 4:** Analysis of the reading times

	Mean (s)	95% CI	*P* value		
			vs. DM	vs. SM	vs. DM + DBT
DM	41.36	19.95–62.77			
SM	42.37	20.96–63.78	0.555		< 0.001
DM + DBT	99.08	77.67–120.48	< 0.001		
SM + DBT	91.87	7.46–113.27	< 0.001	< 0.001	< 0.001

*DM* digital mammograms, *SM* synthesized mammograms, *DBT* digital breast tomosynthesis images, *CI*   confidence interval

## Discussion

The latest version of image processing technology was used to generate a novel SM in this study. The new things about it are as follows. First, the use of this new SM (AI CAD SM) alone increased the reader performance for breast cancer detection in terms of the overall accuracy when compared to DM alone. Second, the reading time was significantly decreased, and the accuracy was improved after using AI CAD SM + DBT when compared to that observed after using DM + DBT. Furthermore, the cancer detection sensitivity was markedly improved after using AI CAD SM alone when compared to that observed after using DM alone (nearly significant at *P* = 0.056). To the best of our knowledge, this is the first study to show that the detection accuracy of the AI CAD SM alone was equivalent to that of DM + DBT. Moreover, the benign recall rates of the AI CAD SM including calcification lesions were lower than those obtained with DM or DM + DBT. One explanation is that mass margins and calcification morphologies are accurately highlighted on AI CAD SM, and this may improve the perception of benign lesions. These results indicated that AI CAD SM maintained the performance benefits of the DBT examinations and could be used as standalone 2D mammograms with higher performance compared to DM alone. Thus, the latest version of the image processing algorithm for generating the AI CAD SM might meet the demand of radiologists.

The AI CAD SM, which was based on the deep convolutional neural network algorithm that processed individual DBT data, presented the suspicious findings that were enhanced by CAD and naturally blended into the corresponding standard AI CAD SM to improve the image quality. That result in the AI CAD SM as concurrent CAD for DBT, which accounts for the significant reduction in the reading time for AI CAD SM + DBT compared to DM + DBT. The reading time for DM alone was 1.01 s shorter than that with AI CAD SM alone (statistical significance notwithstanding). This might be because the readers might not have been able to reach the plateau in their learning curves with regard to the AI CAD SM, despite the training provided before the study. Nonetheless, AI CAD SM alone or in combination with DBT demonstrated a higher performance than DM (alone or DM in combination with DBT).

Evaluation of the performance of the readers based on the breast density is important for screening mammography. In the case of dense breasts, the cancer detection sensitivity of AI CAD SM or AI CAD SM + DBT was better than that of DM or DM + DBT, statistical significance notwithstanding. This finding is in line with those reported previously [12–17]. The sensitivity of AI CAD SM alone in the verified benign cases was significantly lower than that of DM + DBT, which might account for the significantly higher cancer detection accuracy with AI CAD SM when compared to DM. The AI CAD SM generated from DBT data eliminating overlapping breast tissue have power of resistance to dense breasts to easily distinguish malignant lesions and benign lesions. Thus, AI CAD SM should be used proactively in women with dense breasts.

Evaluation of the performance of the readers based on the calcification lesions is important, particularly when using the AI CAD SM.

The sensitivities of DM, AI CAD SM, DM + DBT, and AI CAD SM + DBT were similar for malignant calcification lesions. In the benign calcification lesions, the sensitivities of AI CAD SM alone or AI CAD SM + DBT were significantly lower than those of DM alone or DM + DBT. In addition, the sensitivity of AI CAD SM alone was significantly lower than that of DM + DBT. These findings indicated that AI CAD SM was significantly superior to DM in terms of the specificity and sensitivity for the detection of calcification lesions in a screening setting. Previous studies have reported that the detection and interpretation of calcifications, especially amorphous calcifications, using SM was challenging [13, 18–22]. However, this study showed that the new algorism enhances the contrast of the detail of interest without creating an artifact and suppressing the structure noise thereby improving the visualization of the calcifications on the AI CAD SM. Furthermore, in a diagnostic setting, indeterminate calcifications such as amorphous calcifications are always evaluated using spot compression magnification views to further examine their features during routine clinical practice and to plan their management. Thus, it is important for SM promotion activities not to worry too much for calcification detection and diagnosis. If not using SM in breast imaging, we cannot benefit from the SM faculty forever.

This study has several limitations. First, this study used so an enriched data with malignant cases; hence, the results may not be generalized to a real-world screening program. Second, this study used only the MLO view; access to the CC view could slightly increase the sensitivity and specificity of the results [23]. Thus, further validation using two views might be necessary. Finally, this is a retrospective, single-institution study with a single vendor prototype system comprising cases and radiologists from one country. These study characteristics potentially limit the generalizability of our results for the designing of prospective assessments across a diverse group of radiologists, patients, and institutions. Therefore, the findings of this study should be considered as preliminary results; however, the current SM generated from DBT is already commonly enhanced with suspicious lesions detected by AI CAD [24]. Nevertheless, to the best of our knowledge, this is the first study to assess the reader performance of a new SM and compare it with the original full-field DM when used alone or in combination with DBT in a virtual screening setting.

In conclusion, the use of the AI CAD SM + DBT could allow for a more effective screening program with higher performance, especially in terms of an increase in the detection accuracy and a reduction of in the reading time as well as radiation dose, when compared to the DM + DBT. The AI CAD SM might be considered as standalone 2D mammograms with higher performance compared to the DM.

## Abbreviations

DM: Digital mammograms
AI: Artificial intelligence
CAD: Computer-aided detection
SM: Synthesized mammograms
DBT: Digital breast tomosynthesis
POM: Probability of malignancy
AUC: Areas under the receiver-operating characteristic curve
DCIS: Ductal carcinoma in situ
CC: Cranio-caudal
MLO: Mediolateral oblique
BI-RADS: Breast Imaging Reporting and Data System
ROC: Receiver-operating characteristic
CI: Confidence intervals
2D: Two dimensional
